# High-density scalp EEG data acquired in an inattentional blindness paradigm with background Gestalt stimuli

**DOI:** 10.1016/j.dib.2019.104901

**Published:** 2019-11-29

**Authors:** Alexandre de P. Nobre, Andrey R. Nikolaev, Johan Wagemans

**Affiliations:** a Institute of Psychology, Universidade Federal do Rio Grande do Sul, Porto Alegre, Brazil; b Department of Brain & Cognition, University of Leuven, Leuven, Belgium; c Department of Psychology, Lund University, Lund, Sweden

**Keywords:** Attention, Electroencephalography, Gestalt, Texture segregation, Contour integration, Visual perception, Awareness

## Abstract

Behavioral and electroencephalography (EEG) data were analyzed from 30 participants performing a demanding, attention-capturing task in which they had to detect an occasional decrement of luminance of small discs. Crucially, numerous white segments in the background were presented either in random orientations or configuring a contour shape of a square or a diamond. The participants’ awareness of these background configurations was tested with a questionnaire after the first and second sessions. Based on the questionnaire responses, participants were divided into “aware” and “unaware” groups. These data may be of interest to researchers who wish to understand attentional effects on Gestalt integration processes, as well as luminance discrimination.

**Table:** Specifications Table

Subject	Neuroscience
Specific subject area	Cognitive neuroscience of visual perception and attention. Gestalt perception
Type of data	Raw EEG data Preprocessed behavioral data Questionnaire data Tables
How data were acquired	EEG recorded with a Geodesic Sensor Net with 256 Ag/AgCl scalp electrodes using a high input-impedance Net Amps amplifier (EGI, a Philips company, Eugene, OR, USA). EGI NetStation software was used for recording. Behavioral data were collected using a USB keyboard and a self-report questionnaire.
Data format	Continuous EEG in a raw binary format with markers Tables in the text format Excel tables
Parameters for data collection	Participants were healthy young adults. The data could be analyzed by the factors: Background Gestalt configuration (random, square or diamond); Session of the experiment (1, 2 and 3); Awareness group after the first and second sessions (aware or unaware).
Description of data collection	Data were collected in a dark and electrically shielded room. Participants sat alone in the room wearing a 256-electrodes net. Impedance was below 50 kΩ during recording. Electrode Cz was used as reference, and EEG was recorded at a sampling rate of 250 Hz. Participants performed an experimental task by watching visual stimuli on the screen and responding on the keyboard. The luminance in each trial was adjusted with a QUEST staircase procedure. After each session, they responded to the awareness questionnaire.
Data source location	Institution: KU Leuven - University of Leuven City/Town/Region: Leuven Country: Belgium
Data accessibility	Repository name: Mendeley Data Data identification number: 10.17632/5gv576648z.1 Direct URL to data: https://data.mendeley.com/datasets/5gv576648z/1

**Table:** 

**Value of the Data** • The data are high-density EEG recordings in three different attentional conditions from 30 participants. The dataset allows for any type of EEG analysis, e.g., evoked activity, time-frequency, topographical, source localization, and other analyses that benefit from the large number of EEG channels. • The data allow for the analysis of multiple presentations of preconscious stimuli, which is not often available in inattentional blindness research. Interactions between attention and Gestalt grouping (texture segregation) can be evaluated at a single-trial basis. • The EEG data are accompanied by questionnaire data on awareness of unexpected background stimuli, and may be used to explore relationships between EEG correlates of attention and self-report measures of awareness. • The data were obtained as a conceptual replication of the previous ERP study on unconscious processing of texture segmentation [1], but contain a number of changes and improvements to the stimuli and experimental paradigm. This may be useful for comparison with the original study.

## 1 Data

**Fig. 1:**
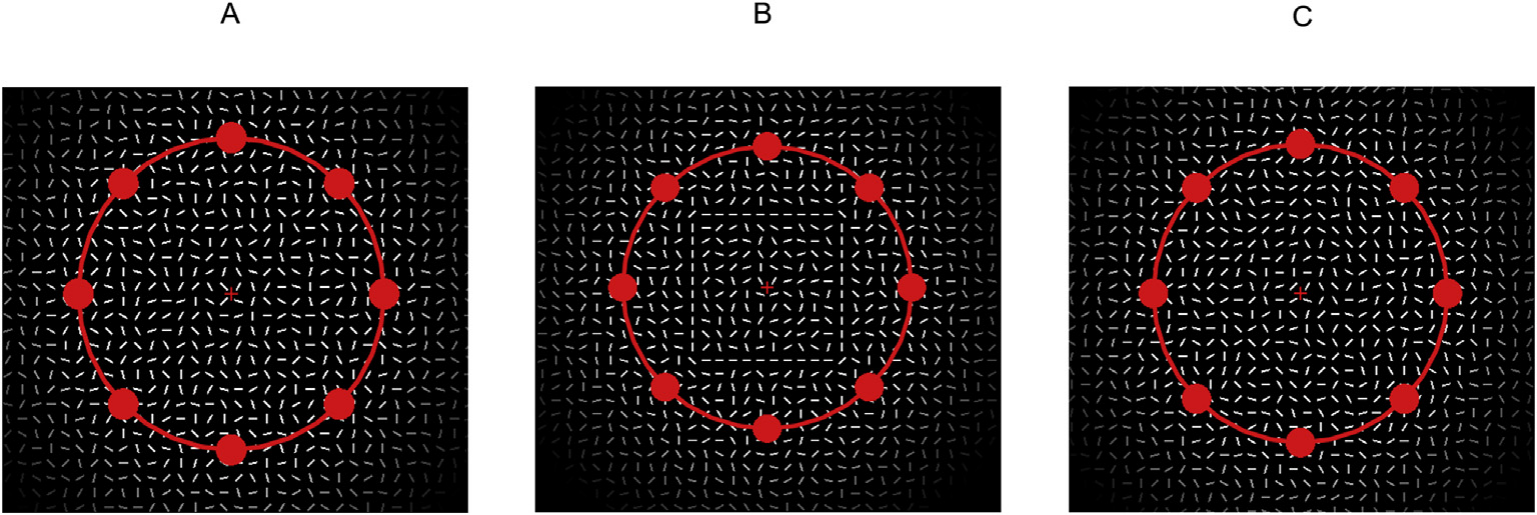
Configurations of the background stimuli. A: random configuration; B: square contour; C: diamond contour.

**Fig. 2:**
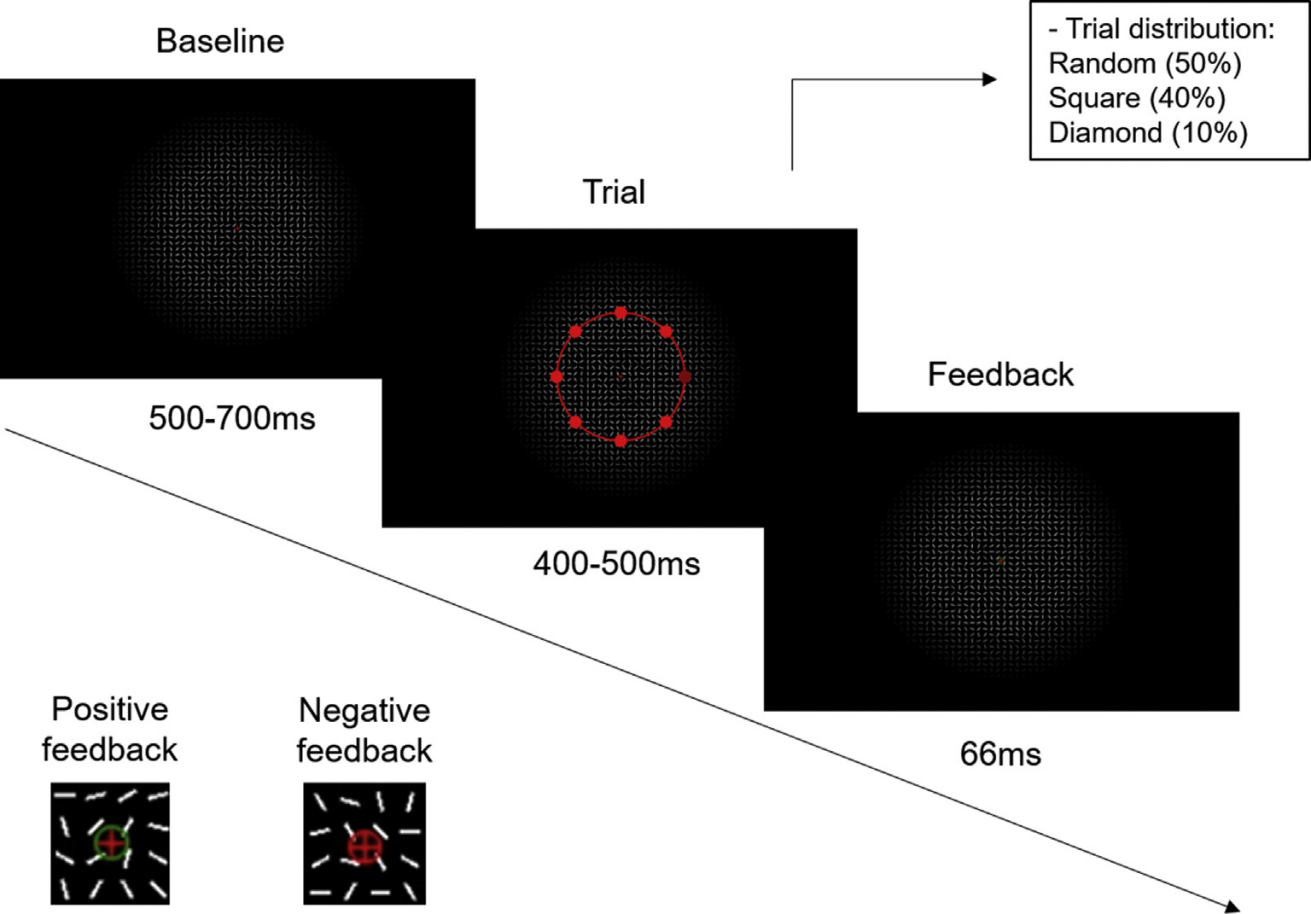
Structure of one trial and examples of positive and negative feedback in the main task following correct and incorrect responses, respectively.

The data consist of EEG recorded continuously during three experimental sessions in 30 participants who performed a detection task in an inattentional blindness paradigm [1]. The corresponding behavioral data consist of RTs and of responses to a questionnaire after Sessions 1 and 2 assessing awareness of the *unexpected stimuli* that were presented in the background concomitantly with the stimuli of the main task. Fig. 1 shows example of stimuli used in each condition of background Gestalt configuration. Fig. 2 shows the trial structure of the experimental paradigm. Table 1 shows means and standard deviations of RTs by background Gestalt configuration, experimental session, and awareness group. Table 2 shows the responses to the awareness questionnaire by experimental session and awareness group: number of reports for each shape (square and diamond), and means and standard deviations of ratings to each item in the confidence ratings and the frequency ratings.

**Table 1:** Means and standard deviations (in parentheses) of RTs by configuration, session, and awareness group, in ms.

Configuration	Group 1		Group 2	
	Session 1	Session 2	Session 1	Session 2
Square	643.24 (43.21)	638.45 (58.50)	651.56 (60.72)	636.24 (41.79)
Random	639.26 (49.77)	638.62 (56.04)	644.30 (44.54)	633.58 (50.67)

**Table 2:** N of participants who reported seeing the square and/or the diamond, and means and standard deviations (in parentheses) for rating responses in the awareness questionnaire by session and group. The remaining participants in Session 1 noticed neither square nor diamond. Note, that the total number of participants in a session is more than 30 because the same participants might notice several shapes.

Group	N of participants who reported each shape			Mean confidence ratings						Mean frequency ratings					
	Square	Diamond	Neither	Diamond	Horizontal Rectangle	X Pattern	Square	Four Squares	Vertical Rectangle	Diamond	Horizontal Rectangle	X Pattern	Square	Four Squares	Vertical Rectangle
Session 1															
Group 1	0	0	13	2.61 (1.32)	1.77 (1.01)	3.08 (1.04)	2.23 (1.48)	2.15 (1.21)	2.15 (1.46)	2.61 (1.32)	1.54 (0.66)	2.54 (1.26)	1.54 (0.66)	1.54 (0.88)	1.61 (0.87)
Group 2	14	6	3	3.35 (1.27)	2.00 (1.17)	3.12 (1.05)	4.17 (1.07)	2.41 (1.12)	2.70 (1.10)	2.82 (1.01)	1.65 (0.70)	3.23 (1.09)	3.47 (1.33)	1.88 (0.78)	2.35 (1.00)
Session 2															
Group 1	13	8	0	4.15 (1.28)	2.15 (1.14)	2.08 (1.32)	4.92 (0.28)	2.15 (1.40)	1.92 (1.19)	2.77 (1.23)	1.54 (0.52)	1.77 (0.83)	4.31 (0.85)	1.61 (0.87)	1.38 (0.51)
Group 2	17	7	0	4.05 (1.43)	1.94 (1.03)	2.59 (0.87)	4.88 (0.33)	1.88 (1.05)	2.47 (1.12)	3.35 (1.22)	1.53 (0.62)	2.06 (1.09)	4.59 (0.51)	1.41 (0.51)	1.76 (0.90)

## 2 Experimental design, materials, and methods

### 2.1 Participants

Data were collected from 37 undergraduate psychology students. Six recordings were excluded due to excess of noise. Another was excluded because the participant misunderstood the awareness questionnaire. The final dataset consists of recordings from 30 (6 male) participants, 18–28 years old (mean = 20.1, SD = 2.09). All participants signed an informed consent form prior to data collection. Ethical approval was provided by the Social and Societal Ethics Committee of the KU Leuven.

### 2.2 Stimuli

We employed stimuli similar to the ones used by Pitts et al. [1], with some changes. A red ring (4.9° of visual angle radius, RGB value of (255, 0, 0), 13.4 cd/m^2^) appeared on a dark (0.07 cd/m^2^) background. Eight discs (1° radius each), matching the ring in color and luminance, were superposed on the ring. The angular distance between any two discs was π/4 radians. At all times, a central fixation cross (0.5°), of the same red color and luminance as the discs, was present at the center of the screen.

Six thousand three hundred white segments (0.34° length, 52.5 cd/m^2^), separated by a distance of 0.46° both vertically and horizontally, were presented in the background. In Pitts et al. [1], white segments were presented at all times, occasionally configuring a 20 × 20 segment pattern shaped as a square or a diamond. We increased the number of segments relative to that in Pitts et al. [1] to fill a larger region of the background and applied a circular inverted Gaussian mask with a radius of 5.65°, so that only a circular region containing 1300 segments in the center was clearly visible.

The white segments were presented in three configurations: random, square, and diamond. In the random configuration, all segments were randomly oriented. In the square and diamond configuration, a subset of the segments configured a square contour (3.5°× 3.5°) or a diamond contour (3.2°× 3.2°) centered at the fixation cross, while the remaining segments were randomly oriented.

### 2.3 Procedure

The experiment was conducted in an electrically shielded, dark room. Participants sat 57 cm away from a 14-inches LED screen with a 60 Hz refresh rate. The experiment was run using the Psychopy software [2]. Before the experiment, during instructions, participants were shown an example of a trial consisting of a screenshot of the red ring and discs superposed on the white segments, which were randomly oriented (Fig. 1A). If participants asked about the purpose of the white segments, they were told that the segments create a visual noise to make the task harder.

The trial structure is depicted in Fig. 2. Each trial started with a baseline period that had a duration varying randomly according to a uniform distribution in the interval between 500 and 700 ms. During the baseline period, the white segments were presented in random orientations along with the red fixation cross; neither the disc nor the rings were present. Once every second, the ring and the discs appeared on the screen, remaining visible for a period between 400 and 500 ms (a trial period). In the design employed by Pitts et al. [1], a trial had a fixed duration of 300 ms. Because with such duration the offset of the trial coincided with the onset time of the Nd2 effect observed by Pitts et al. [1], we presented the stimulus for a longer duration. Additionally, to avoid confounds with the offset of the stimulus, we introduced a jitter of 100 ms in the stimulus duration. During the trial period, the white background segments formed the unexpected stimulus in 50% of trials: a square contour (40% of trials; Fig. 1B) or a diamond contour (10% of trials; Fig. 1C). In the remaining 50% of trials, the segments were randomly oriented. To reduce the likelihood that participants might perceive the background configurations because they had not yet begun to focus on the task, in the first trial of each block the white segments were always configured randomly. The orientation of the random segments changed in each trial.

In Pitts et al.‘s [1] design, an attention-capturing manipulation consisted of the ring and discs rotating either clockwise or counterclockwise in each trial and returning to their original position during the baseline period. We replaced the rotation by another attention-capturing manipulation, consisting of the appearance and disappearance of the ring and the discs for the duration of the trial. The task-relevant event in this experiment was the same as in Pitts et al.‘s [1]: a decrease in the luminance of one of the disks. In target-absent trials, all discs had the same luminance. In target-present trials, the luminance of one of the discs, chosen randomly from the eight discs, was decreased for the duration of a trial. The participants' task was to press a space bar with their index finger when they notice a darker disc, as fast and accurately as they could. They received feedback (see the inset in Fig. 2) as a green circle of 0.22° around the fixation cross for correct responses, when a target was presented and a response was emitted; or a red circle for misses, when a target was presented but no response was emitted, as well as for false alarms, when no target was presented and a response was emitted. The feedback was presented immediately after the response, or after the trial period in target-present trials in case of misses. The feedback remained on-screen for 66 ms. No feedback was presented for correct rejections (when no target was presented and no response was emitted).

To keep task difficulty constant in the course of the experiment, the luminance decrement of each trial was determined using a QUEST staircase procedure [3]. The QUEST is an adaptive procedure that employs prior information to efficiently fit a Weibull psychometric function to the data in each trial; this psychometric function is then used to determine stimulus intensity. In each trial, the red component of the disc's RGB value was changed, varying between 0 and 255, in such a way determining stimulus intensity. The QUEST computed the detection threshold using data from target-present trials to arrive at a value corresponding to 50% correct performance. The QUEST was used across all sessions of the experiment.

The experiment was divided into four sessions: a practice and three test sessions. The practice session comprised five blocks of 61 trials each, for a total of 305 trials. Each test session comprised 10 blocks of 61 trials each, for a total of 610 trials per session, 10% of which were target-present trials. Between blocks, participants had self-paced pauses. They started the next block by pressing the space bar. During practice, no background contour shapes were presented. Participants were not informed that the first session was actually a practice session, to avoid encouraging participants from becoming more alert or focused in Session 1, which might have increased the likelihood of perceiving the background shapes. The practice session is not a part of the dataset.

The session structure reproduced a typical inattentional blindness design [4], which includes an “inattention” phase, a “divided attention” phase and a “full attention” phase. The first session corresponded to the “inattention” phase. After the first session, participants were asked to answer a questionnaire about the unexpected stimuli (Appendix). This questionnaire consisted of a yes-or-no question and a free recall question (to which participants could answer by writing or drawing) about the unexpected stimulus. The questionnaire also included two rating scales. Additionally, we included a question inquiring during which block within the session participants noticed the unexpected stimuli, for those who reported seeing it. After the questionnaire, the second session was started, which corresponded to the “divided attention” phase. After the second session, participants answered the same questionnaire again. In the last session, the participants’ task changed: they were asked to ignore the discs and to attend to the background patterns instead. The task was to respond to the appearance of the diamond pattern. The last session corresponded to the “full attention” phase.

### 2.4 Electrophysiological recordings

EEG data were collected using a Geodesic Sensor Net with 256 Ag/AgCl electrodes and the high input impedance amplifier (EGI, a Philips company, Eugene, OR, USA). The EGI net included electrodes for registration of horizontal and vertical electrooculograms (EOGs). Data were digitized at a sampling rate of 250 Hz. Impedance was kept below 50 kΩ. During recording, all electrodes were referenced to the vertex electrode (Cz). EEG was filtered on-line using a low-frequency cutoff of 0.1 Hz and a high-frequency cutoff of 100 Hz. The raw data for all recordings are available in a public repository, in addition to data for the behavioral task and the awareness questionnaires (https://data.mendeley.com/datasets/5gv576648z/1).

### 2.5 Behavioral analysis

For the main task, we registered RTs and luminance decrements resulting from the adaptive procedure. Trials with RTs above or below three standard deviations from the mean were excluded as outliers. All participants who answered “yes” to the yes-or-no question of the questionnaire were considered aware of the unexpected stimuli, and responses to question 3 (free recall) were inspected to determine which shapes they noticed. Participants who answered “no” to the yes-or-no question were judged aware or unaware according to their response in the rating scales: If they rated three or less in both the confidence rating and the frequency rating, they were considered unaware. Conversely, if they rated four or more in either the confidence rating or the frequency rating, they were considered aware.

#### 2.5.1 Behavioral data

Table 2 shows the data for the awareness questionnaire. Using the criteria described above to classify participants as aware or unaware, we divided the participants into two groups based on their answers to the questionnaire after Session 1: Group 1 was composed of participants who were considered unaware (by answering “no” to the yes-or-no question and rating their confidence/frequency of seeing the square as 3 or less), while Group 2 was formed by participants who were considered aware (by answering “yes” to the yes-or-no question and rating their confidence/frequency of seeing the square as 4 or 5). Based on the responses, 13 participants were included in Group 1, and 17 were included in Group 2. We also examined responses to the free recall question (question 3) to determine which participants reported seeing the square and/or the diamond by either drawing or describing the shapes. All participants from both groups noticed the square in Session 2.
