# Supplementary material for: High-density scalp EEG data acquired in an inattentional blindness paradigm with background Gestalt stimuli
Source: Data Brief. 2019 Nov 29;28:104901. doi: 10.1016/j.dib.2019.104901 (PMC6920440; doi:10.1016/j.dib.2019.104901)
Supplement: Multimedia component 1 [file mmc1.docx]

**Appendix**

Post-phase questionnaire

Phase:_____ Subject:______

Date:____________

1. During this session, did you notice any patterns within the little white lines in the background?

| Yes | No |
| --- | --- |

1. If you did notice any patterns, at which moment during this session did that first happen?

During blocks 1 to 3

During blocks 4 to 7

During blocks 8 to 10

1. If you did see any patterns, please describe (or draw) what you saw in as much detail as possible.

* For the items below, the experimenter will provide examples on the computer screen.

1. Rate how confident you are that you saw each pattern during the experiment.

| Please use the following scale: | Diamond | 1 | 2 | 3 | 4 | 5 |
| --- | --- | --- | --- | --- | --- | --- |
| 1 – very confident I did *not* see it  2 – confident I did *not* see it  3 – uncertain  4 – confident I saw it  5 – very confident I saw it | Horizontal rectangle | 1 | 2 | 3 | 4 | 5 |
|  | X pattern | 1 | 2 | 3 | 4 | 5 |
|  | One big square | 1 | 2 | 3 | 4 | 5 |
|  | Four small squares | 1 | 2 | 3 | 4 | 5 |
|  | Vertical rectangle | 1 | 2 | 3 | 4 | 5 |

1. Estimate how often you saw each pattern during the experiment.

| Please use the following scale: | Diamond | 1 | 2 | 3 | 4 | 5 |
| --- | --- | --- | --- | --- | --- | --- |
| 1 – never  2 – rarely/less than 10 times  3 – infrequently/10-50 times  4 – frequently/50-100 times  5 – very frequently/more than 100 times | Horizontal rectangle | 1 | 2 | 3 | 4 | 5 |
|  | X pattern | 1 | 2 | 3 | 4 | 5 |
|  | One big square | 1 | 2 | 3 | 4 | 5 |
|  | Four small squares | 1 | 2 | 3 | 4 | 5 |
|  | Vertical rectangle | 1 | 2 | 3 | 4 | 5 |
